# Are peptide conjugates the golden therapy against obesity?

**DOI:** 10.1530/JOE-18-0264

**Published:** 2018-05-30

**Authors:** S J Brandt, M Kleinert, M H Tschöp, T D Müller

**Affiliations:** 1 Institute for Diabetes and ObesityHelmholtz Diabetes Center, Helmholtz Zentrum München, German Research Center for Environmental Health (GmbH), Neuherberg, Germany; 2 German Center for Diabetes Research (DZD)Neuherberg, Germany; 3 Division of Metabolic DiseasesTechnische Universität, Munich, Germany

**Keywords:** peptides, diabetes, obesity, metabolism

## Abstract

Obesity is a worldwide pandemic, which can be fatal for the most extremely affected individuals. Lifestyle interventions such as diet and exercise are largely ineffective and current anti-obesity medications offer little in the way of significant or sustained weight loss. Bariatric surgery is effective, but largely restricted to only a small subset of extremely obese patients. While the hormonal factors mediating sustained weight loss and remission of diabetes by bariatric surgery remain elusive, a new class of polypharmacological drugs shows potential to shrink the gap in efficacy between a surgery and pharmacology. In essence, this new class of drugs combines the beneficial effects of several independent hormones into a single entity, thereby combining their metabolic efficacy to improve systems metabolism. Such unimolecular drugs include single molecules with agonism at the receptors for glucagon, glucagon-like peptide 1 and the glucose-dependent insulinotropic polypeptide. In preclinical studies, these specially tailored multiagonists outperform both their mono-agonist components and current best in class anti-obesity medications. While clinical trials and vigorous safety analyses are ongoing, these drugs are poised to have a transformative effect in anti-obesity therapy and might hopefully lead the way to a new era in weight-loss pharmacology.

## Introduction

Obesity is a devastating condition of pandemic dimensions. In 2015, there were 107.7 million obese children and 603.7 million obese adults worldwide (Afshin *et al.* 2017), and this number is expected to rise. Overweight and obesity are associated with a number of comorbidities, most importantly type 2 diabetes (T2DM), cardiovascular disease, hypertension, dyslipidemia and several kinds of cancer, predominantly gastrointestinal (GI). In 2015, around 4 million deaths were attributed to overweight and obesity (Afshin *et al.* 2017).

Hypothetically speaking, obesity could be prevented simply by reducing food intake and increasing physical activity. However, adherence to lifestyle interventions such as regular exercise is poor. A number of psychological and economic factors are involved in such compliance, and humans might be evolutionarily predisposed to a positive energy balance (Wells 2006). Furthermore, once excess weight has been gained, human metabolism intrinsically defends against its loss (MacLean *et al.* 2015).

Since lifestyle interventions have so far proven insufficient to combat our obesity pandemic, other interventions are needed. To date, the most effective and long-lasting intervention is bariatric surgery. Of the various types of bariatric surgeries available, Roux-en-Y gastric bypass and biliopancreatic diversion/duodenal switch surgeries are the most common and successful, with reported initial excess weight reduction of up to 68–70%, where excess weight is defined as the difference between total preoperative weight and ideal weight (Buchwald 2002, Buchwald *et al.* 2004). Despite unquestionable effectiveness, bariatric surgery is typically only available to a small subset of individuals, with inclusion criteria being a BMI greater than 40 or greater than 35 with a comorbidity such as diabetes or heart disease (1992). In addition, the surgery itself is costly and not without risk (Chang *et al.* 2014).

Notably, improvement of glycemic control by bariatric surgery is rapid and is often observed even before a clinically relevant weight loss (Pories *et al.* 1995, Peterli *et al.* 2009, Bayham *et al.* 2012). Despite intense scientific investigation, changes in metabolic rate or intestinal nutrients absorption do not seem to explain the efficacy and sustainability in weight reduction (Olbers *et al.* 2006, Odstrcil *et al.* 2010, Carswell *et al.* 2014, Munzberg *et al.* 2015, Schmidt *et al.* 2016). Changes in food intake are frequently reported after bariatric surgery and are commonly considered a causal factor for the weight loss (Brolin *et al.* 1994, Sjostrom *et al.* 2004, Laurenius *et al.* 2012, Munzberg *et al.* 2015). Notably, such differences in food intake do not seem to rely on physical limitations of the GI tract (Ryan *et al.* 2014), but rather result from changes in food preference, taste perception and modifications in the central food reward system (Scruggs *et al.* 1994, Burge *et al.* 1995, Miras & le Roux 2010, Shin & Berthoud 2011, Mathes & Spector 2012, Laurenius *et al.* 2013). It seems fair to hypothesize that such changes are likely mediated via neuronal and/or humoral factors (Clemmensen *et al.* 2017). For example, following Roux-en-Y gastric bypass, gastric banding or sleeve gastronomy, there is an increase in the secretion of glucagon-like peptide 1 (GLP-1) (Laferrere 2016, Meek *et al.* 2016, Clemmensen *et al.* 2017), which is known not only for its beneficial effects on glycemia but also for its ability to decrease body weight via CNS-induced inhibition of food intake (Sisley *et al.* 2014).

**Figure 1:**
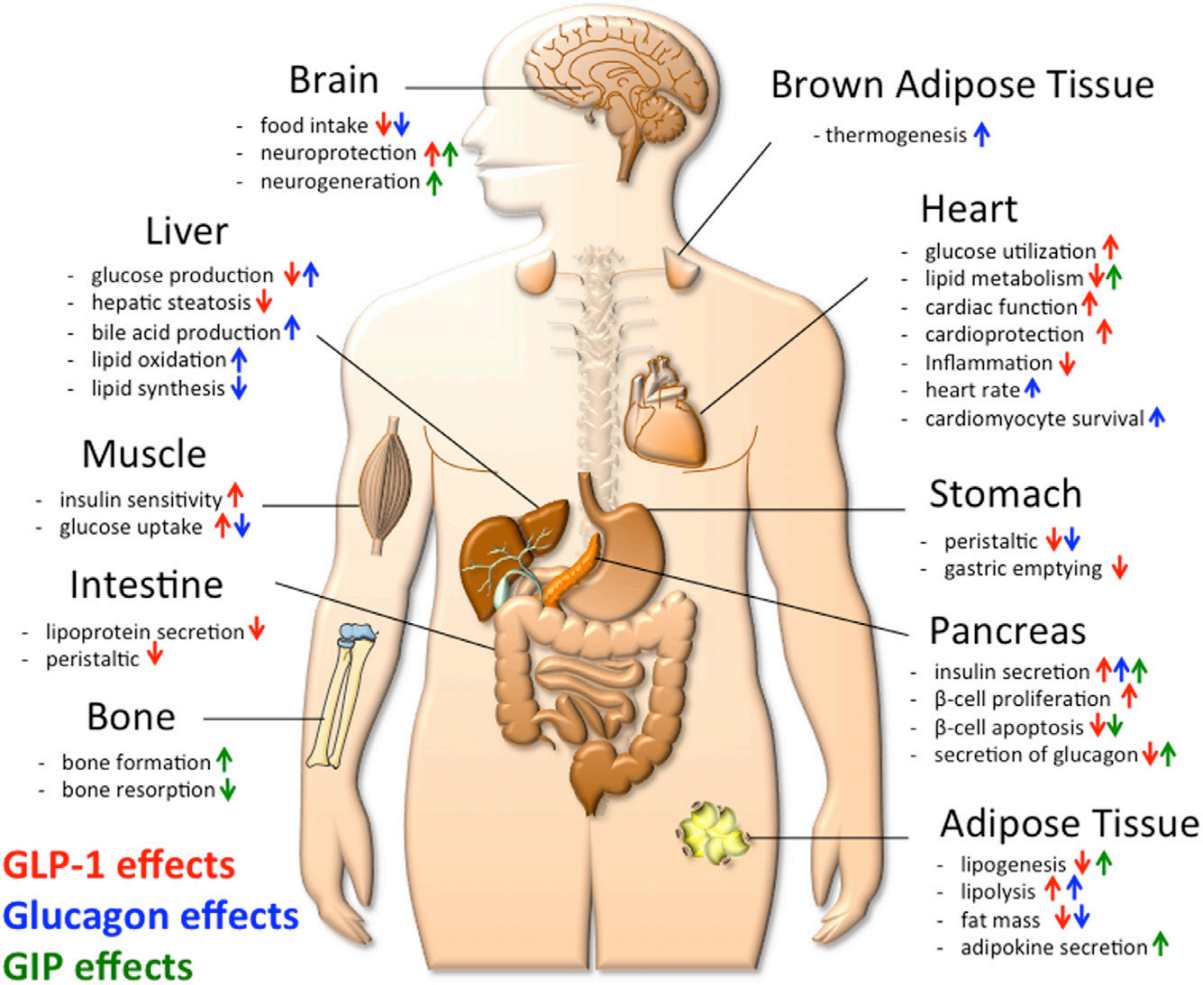
Schematic demonstrating the qualitative metabolic effects of GLP-1 (red arrows), glucagon (blue arrows) and GIP (green arrows) on systems metabolism, including key metabolic tissues. Arrows pointing upwards indicate an increase or improvement, while arrows pointing downwards indicate a decrease.

GLP-1 is secreted by the intestinal L-cells in response to nutrient stimuli. GLP-1 directly acts on the β-cells to increase glucose-stimulated insulin secretion and also through the central nervous system to decrease food intake (Fig. 1) (Muller *et al.* 2017). Native GLP-1 is rapidly degraded by dipeptidyl peptidase IV (DPP-IV), which cleaves native GLP-1 at the N-terminal alanine at the second position, resulting in the generation of the inactive GLP-1_9–36amide_ or GLP-1_9–37_ (Mentlein *et al.* 1993, Deacon *et al.* 1995, Kieffer *et al.* 1995). Native GLP-1 accordingly has a circulating half-life of 1.5–5 min (Hui *et al.* 2002, Baggio & Drucker 2007). Modifications to the native GLP-1 sequence have overcome this limitation. Common modifications include the substitution of a d-Serine or aminoisobutyric acid (Aib) residue at position 2 to increase resistance to peptidase degradation. Another common modification is extension of the peptide to include the nine amino acid C-terminal extension (CEX) of exendin-4, which stabilizes the secondary structure and can (depending on the peptide) improve glucagon receptor agonism (Neidigh *et al.* 2001, Li *et al.* 2007, Chabenne *et al.* 2010, Finan *et al.* 2013, 2015*a*,*b*). Additional modifications such as site-specific acylation or conjugation with large biomolecules have resulted in a series of commercially available GLP-1 analogs, with varying efficacies (Finan *et al.* 2015*a*,*b*). Despite the development of several iterations, these GLP-1 analogs only have modest weight-lowering efficacy, which, depending on dose and duration of treatment, typically fall in the range of 1–5 kg (Bush *et al.* 2009, Nauck *et al.* 2009, 2016, Bergenstal *et al.* 2010, Fonseca *et al.* 2012, Meier 2012, Russell-Jones *et al.* 2012, Rosenstock *et al.* 2013, Woodward & Anderson 2014, Davies *et al.* 2015, Lau *et al.* 2015, Thompson & Trujillo 2015, Dungan *et al.* 2016, Pratley *et al.* 2018). Side effects such as nausea and GI distress preclude higher doses to drive greater weight loss. Therefore, it is clear that while GLP-1 analogs are beneficial to improve glycemia, targeting solely the GLP-1 receptor for the purpose of lowering body weight has limitations.

Serendipitously, native GLP-1 shows high sequence homology to glucagon and the glucose-dependent insulinotropic polypeptide (GIP). High sequence homology is also present in the receptors for GLP-1, glucagon and GIP, which together makes these peptides inherently prone to sequence hybridization for the purpose of simultaneously activating their receptors with only one molecule. Notably, glucagon can decrease body weight via inhibition of food intake and elevation of energy expenditure (Muller *et al.* 2017). Consequently, it was believed that such a single molecule with dual agonism at the receptors for glucagon and GLP-1 would lead to complementary (and ideally synergistic) pharmacological action, putatively driving greater weight loss and glycemic benefits through non-redundant signaling pathways. Any observed beneficial action would naturally create hope for the possibility of lower dosing schemes, thus potentially reducing the possibility of side effects, such as those typically seen at high doses of GLP-1.

The unimolecular formulation has several advantages compared to the loose adjunct administration of the single peptides. The key biological difference is that each independent peptide would have its specific and potentially unique pharmacokinetic profile. Accordingly, the peptides in such a loose combination would likely differ in their rates of absorption, distribution, metabolism and clearance. In contrast, a unimolecular multiagonist would have only one pharmacokinetic profile, which was hypothesized to result in superior metabolic benefits compared to a loose co-mixture of the single peptides. Furthermore, in terms of practicality, a single molecule polyagonist can more easily achieve regulatory approval.

## GLP-1/glucagon co-agonism

**Figure 2:**
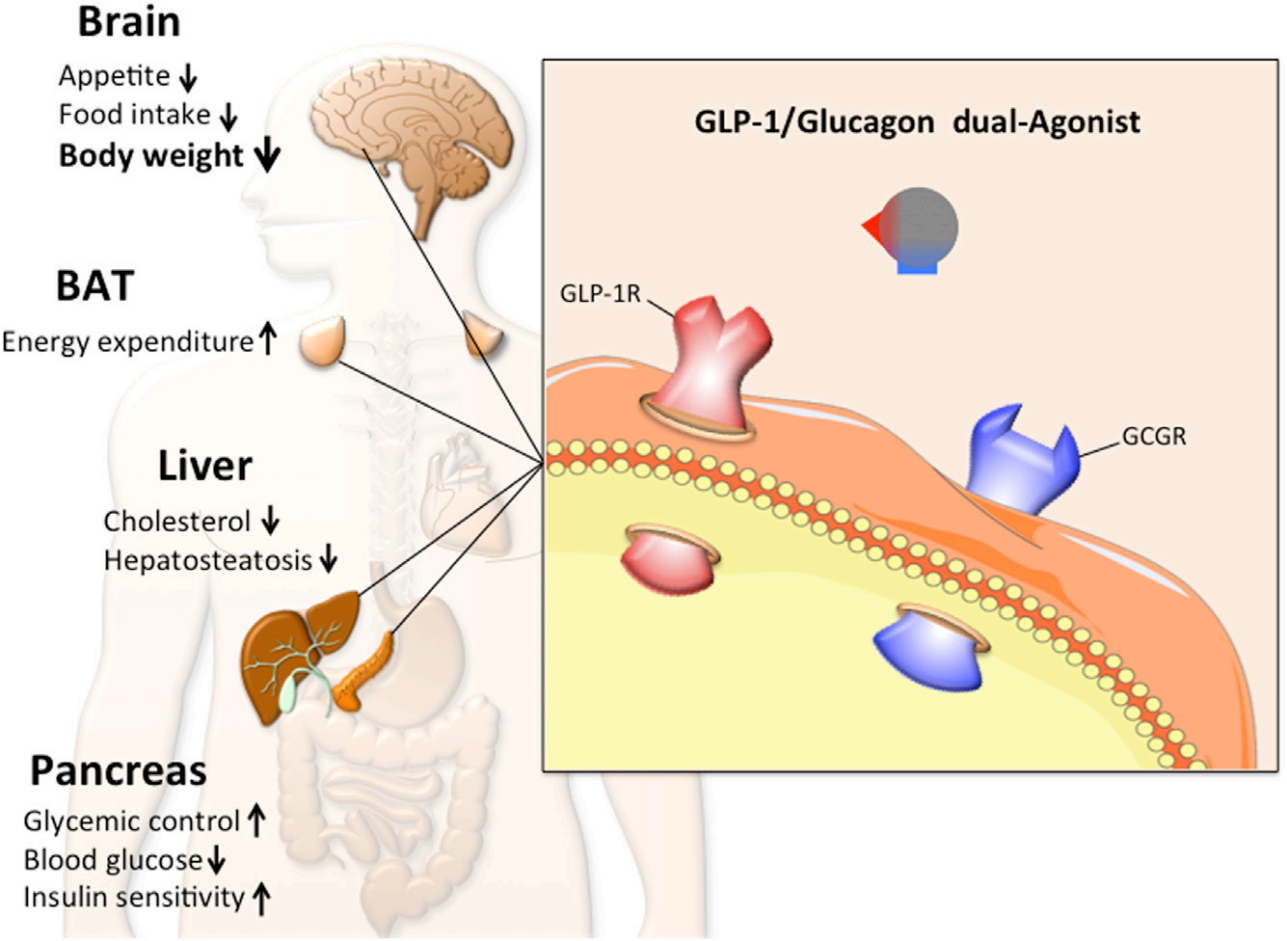
Schematic demonstrating the working principle, metabolic effects and key target tissues of the GLP-1/glucagon dual agonist, with the size of the text weighted to indicate the magnitude of the observed effect. Arrows pointing upwards indicate an increase or improvement, while arrows pointing downwards indicate a decrease. This dual agonist most prominently affects body weight.

The combination of GLP-1R and glucagon receptor (GCGR) agonism into a single entity seems, at first glance, counter-intuitive. Glucagon raises blood glucose levels by stimulating gluconeogenesis and glycogenolysis (Fig. 1) (Muller *et al.* 2017). In an obese patient, for whom diabetes is a liability or comorbidity, raising blood glucose would obviously be undesirable. Glucagon has indeed been postulated to play a key role in the development of type 2 diabetes (Unger & Cherrington 2012) and patients with T2DM are frequently reported to have postprandial hyperglucagonemia due to impaired glucose inhibition of glucagon secretion (Muller *et al.* 1970, Unger *et al.* 1970, Gerich *et al.* 1976, Felig *et al.* 1978, Butler & Rizza 1991, Kelley *et al.* 1994). However, glucagon also increases satiety after a meal and increases energy expenditure in rodents and humans (Muller *et al.* 2017). The logic behind a dual agonist targeting the receptors for GLP-1 and glucagon was thus that the insulinotropic effects of GLP-1 would buffer against any hyperglycemic liability of glucagon, while the anorectic effect of GLP-1 would synergize with glucagon’s anorectic and thermogenic effects to ultimately drive weight loss (Fig. 2). One can argue that mother nature developed the first of such GLP-1/glucagon dual-agonists with oxyntomodulin (OXM). Notably, however, despite having activity at both cognate receptors, OXM greatly favors GLP-1R over GCGR (Pocai 2014).

The first patented and preclinically evaluated GLP-1/glucagon dual agonist was developed by the groups of Richard DiMarchi and Matthias Tschöp. The molecule is based on the glucagon sequence, with key GLP-1 residues introduced to impart GLP-1R agonism (Day *et al.* 2009). This dual agonist also includes an Aib residue at position 2 to protect from DPP-IV cleavage. A 40 kDa PEGylation was added on cysteine 24 to prolong *in vivo* action, and a lactam bridge between Glu16 and Lys20 was introduced to stabilize the secondary structure of the molecule and to boost GCGR activity (Day *et al.* 2009). In DIO mice monitored for 7 days, a single injection of 325 nmol/kg resulted in a decrease in food intake and a body weight loss of 25%, primarily due to a loss of fat mass (Day *et al.* 2009). In a more chronic setting, weekly administration of 70 nmol/kg of the co-agonist for 1 month resulted in a 28% decrease in body weight, primarily fat mass, as well as an improvement in glucose tolerance, an increase in energy expenditure and an increase in the utilization of lipids as energy substrates (Day *et al.* 2009). A 27-day study of the same dose revealed that the co-agonist decreases plasma triglycerides, LDL cholesterol and total cholesterol, decreased circulating leptin and normalized liver lipid content (Day *et al.* 2009). These preclinical results demonstrated the multifaceted ‘approach’ of the co-agonist, which robustly corrects obesity and improves multiple aspects of metabolism simultaneously.

Another example of a GLP-1R/GCGR co-agonist was developed by the research group of Merck. This co-agonist was inspired by the native hormone OXM. In order to boost the activity and efficacy of OXM, d-Serine was substituted at position 2 and a cholesterol moiety was added to the C-terminus of the peptide (Pocai *et al.* 2009). The resulting DualAG peptide showed nearly balanced potency at the receptors for GLP-1 and glucagon (Pocai *et al.* 2009). In DIO mice, every-other-day subcutaneous injections of 1.9 µmol/kg of DualAG for 14 days resulted in a 30% reduction in food intake and a 25% body weight loss, primarily due to a loss of fat mass (Pocai *et al.* 2009). In addition, DualAG induced significant improvements in glucose tolerance and normalized blood glucose levels, benefits that are likely secondary to the loss of body weight (Pocai *et al.* 2009). These effects were blunted in either GLP-1R^−/−^ or GCGR^−/−^ mice (Pocai *et al.* 2009), demonstrating the contribution of both receptors to the metabolic effects and emphasizing the importance of dual agonism for synergistic effects.

A third example of a GLP-1R/GCGR co-agonist has been developed by Sanofi. This peptide is based on the exendin-4 sequence with additional glucagon residues introduced to enhance activity at the GCGR (Evers *et al.* 2017). Like many of the other dual-agonists, this peptide incorporated a d-Serine at position 2 to reduce peptidase degradation, and a palmitic acid at a Lys14 to extend the half-life, which was measured to be 3.2 h in healthy mice (Evers *et al.* 2017). In DIO mice, a twice-daily subcutaneous injection of 50 µg/kg of this dual agonist over the course of 33 days resulted in a 29.1% drop in body weight, greater than the 13.6% drop in body weight from a matched dose of liraglutide (Evers *et al.* 2017). Similarly, in db/db mice, twice-daily subcutaneous injections of 50 µg/kg of the dual agonist over the course of 32 days resulted in lower HbA1c levels than control animals (Evers *et al.* 2017).

A fourth GLP-1/GCGR co-agonist (MEDbib382) is under development by MedImmune. This peptide has balanced activity at both receptors and increased stability against peptide degradation (Henderson *et al.* 2016). The half-life of this dual agonist is further enhanced by palmitoylation at Lys10, which promotes binding to serum albumin. In DIO mice, acute administration of 10 nmol/kg suppresses food intake and improves glucose tolerance, although these effects are absent in GLP-1R knockout mice (Henderson *et al.* 2016). In a more chronic setting, a daily dose of 30 nmol/kg of MEDbib382 results in a 30% decrease in body weight and suppression of food intake over the course of 4 weeks (Henderson *et al.* 2016). In a separate study, 3 weeks of 10 nmol/kg resulted in a greater weight loss than pair fed controls, and an increase in oxygen consumption and decrease in the respiration exchange ratio compared to vehicle controls, all without a difference in locomotor activity (Henderson *et al.* 2016), suggesting an energy expenditure component to the observed weight loss. Importantly, the weight-loss effects of MEDbib382 translate into cynomolgus monkeys. In a 29-day study with doses between 8 and 27 nmol/kg MEDbib382, cynomolgus monkeys dose dependently lost between 5 and 13% of their body weight (Henderson *et al.* 2016). This weight loss was accompanied by a reduction in food intake (Henderson *et al.* 2016). After treatment cessation, monkeys that had been treated with MEDbib382 stabilized at a lower body weight than the control monkeys (Henderson *et al.* 2016), perhaps indicating that MEDbib382 induced a lower ‘set point’ for body weight maintenance. In a separate study, 29 days of administration of 4–27 nmol/kg in cynomolgus monkeys did not affect blood glucose (Henderson *et al.* 2016).

These are just some of the GLP-1R/GCGR coagonists currently in development, and several of these peptides have progressed to Phase I and Phase II clinical testing (Table 1). Undoubtedly, more information on the clinical effects of these drugs will be available soon.

**Table 1:** Multiagonists in development.

Target receptors	Drug	Company	Status
GLP-1R/GCGR	HM12525A	Hamni Pharmaceuticals	Phase II
	JNJ-54728518	Janssen Pharmaceuticals	Phase I
	MEDbib382	MedImmune	Phase II
	MK-8521	Merck	Phase II
	NN9277	Novo Nordisk	Phase I
	MOD-6030/1	Prolor/OPKO Biological	Preclinical
	SAR425899	Sanofi	Phase II
	VPD-107	Spitfire Pharma	Preclinical
	TT-401	Transition Therapeutics	Phase II/not advancing
	ZP2929	Zealand	Phase I
GLP-1R/GIPR	CPD86	Eli Lilly	Preclinical
	LY3298176	Eli Lilly	Phase II
	NN9709/MAR709/RG7697	Novo Nordisk/Marcadia	Phase II
	SAR438335	Sanofi	Phase I
	ZP-I-98	Zealand	Preclinical
	ZP-DI-70	Zealand	Preclinical
GLP-1R/GCGR/GIPR	HM15211	Hamni Pharmaceuticals	Preclinical
	MAR423	Novo Nordisk/Marcadia	Phase I

### GLP-1/GIP co-agonism

GIP is a 42 amino acid protein secreted by the enteroendocrine K-cells of the proximal small intestine in response to nutrient intake (Drucker 2006). As an incretin hormone, the primary role of GIP is to stimulate insulin secretion (Fig. 1). Treatment with GIP is reported to normalize blood glucose and to improve glucose tolerance (Hinke *et al.* 2002, Gault *et al.* 2011, Kim *et al.* 2012), although its insulinotropic effects are blunted in some individuals with type 2 diabetes (Vilsboll *et al.* 2002). Despite its glycemic benefits, GIP was dismissed as a potential anti-obesity target due to some reports testifying GIP is obesogenic in nature in mice and certain cell lines (Eckel *et al.* 1979, Oben *et al.* 1991, Miyawaki *et al.* 2002, McClean *et al.* 2007, Althage *et al.* 2008, Gogebakan *et al.* 2012, Finan *et al.* 2016). However, more recent studies demonstrate that chronic treatment with GIP can decrease body weight in rodents (Finan *et al.* 2016). Mice overexpressing GIP show improved glycemic control and resistance to diet-induced obesity (Kim *et al.* 2012). Chronic GIPR agonism further improves glucose metabolism in DIO mice without signs of excess weight gain (Martin *et al.* 2013). Transgenic pigs expressing a dominant negative GIP receptor in the pancreas also show impaired glucose tolerance due to delayed insulin secretion, impaired insulinotropic action of GIP, roughly 60% reduced β-cell proliferation and reduced islet mass of up to 58% at the age of 1 year (Renner *et al.* 2010).

**Figure 3:**
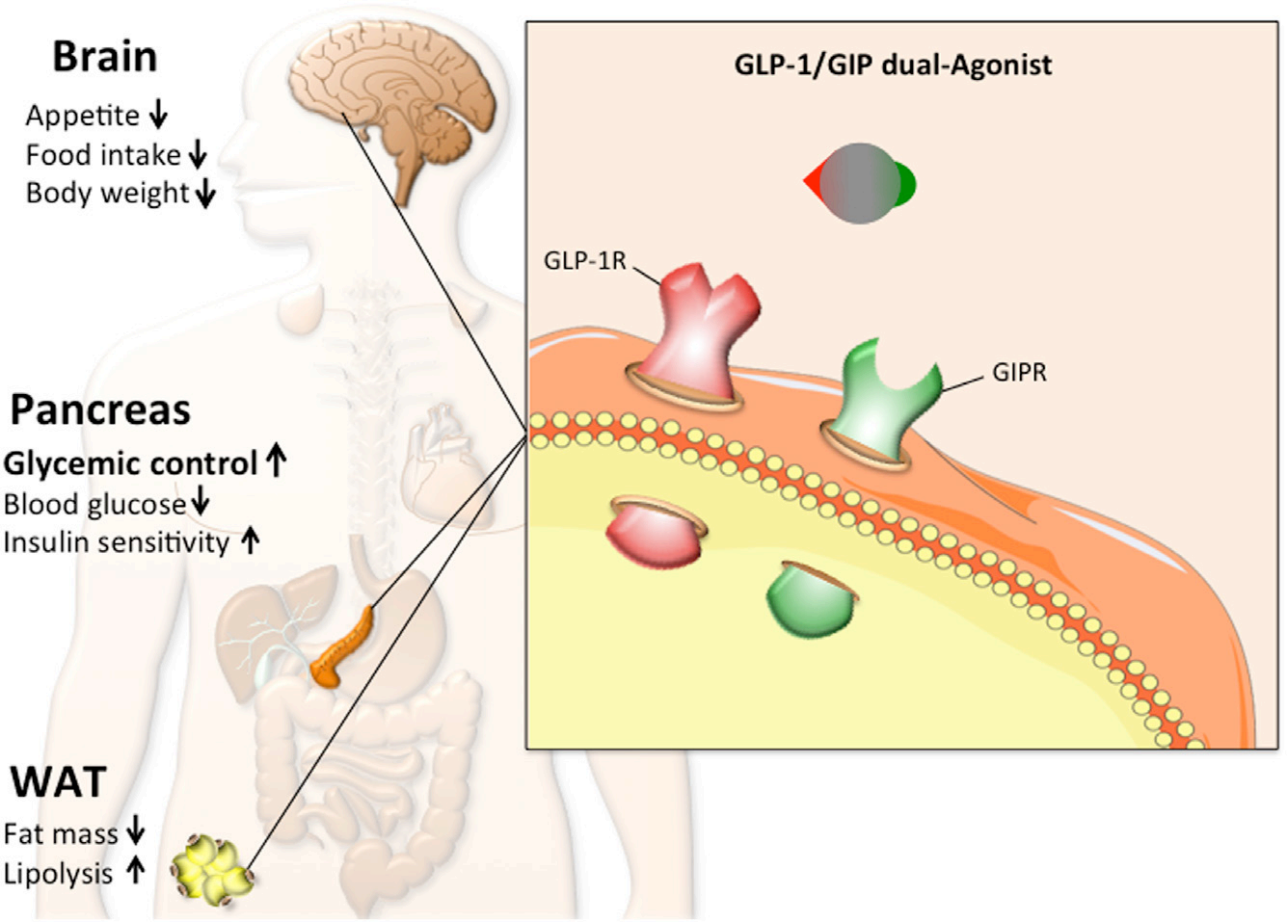
Schematic demonstrating the working principle, metabolic effects and key target tissues of the GLP-1/GIP dual agonist. Arrows pointing upwards indicate an increase or improvement, while arrows pointing downwards indicate a decrease. The emphasis on glycemic control indicates the relative magnitude of the effect.

The rationale to combine the pharmacology of GLP-1 and GIP in a single entity was based on the hypothesis that such a dual incretin hormone action would maximize the glycemic benefits while the anorexigenic effect of GLP-1 would restrain any obesogenic potential of GIP (Fig. 3). In support of this hypothesis, co-administration of GLP-1 and GIP in mice was *a priori* confirmed to improve glycemia and body weight loss in DIO mice (Finan *et al.* 2013).

Two unimolecular dual incretin (‘twincretin’) hormones were subsequently created based on the primary glucagon sequence. The dual-agonists incorporated key GLP-1 and GIP residues such that the peptide activated both the GLP-1R and GIPR with equal potency *in vitro* (Finan *et al.* 2013). Other modifications included an Aib residue at position 2 to increase resistance to DPP-IV cleavage. This peptide was either acylated with a C16:0 fatty acid (acylated version) at Lys40 or PEGylated with 40 kDa PEG at Cys24 (PEGylated version) to prolong *in vivo* action. The C-terminal ends of the peptides were further modified to carry the CEX tail from exendin-4. Daily administration of 30 nmol/kg of the unacylated version of the dual agonist in DIO mice over the course of 7 days resulted in a 14% drop in body weight, greater than a comparable dose of exendin-4 (Finan *et al.* 2013). A single 30 nmol/kg dose of the 16-carbon acylated version of the peptide resulted in an 18.8% body weight drop (Finan *et al.* 2013). Both versions of the peptide decreased food intake, lowered body weight primarily through the loss of fat mass and decreased blood glucose levels (Finan *et al.* 2013). The PEGylated version of the peptide yielded similar results with less frequent dosing (Finan *et al.* 2013). Like the GLP-1R/GCGR co-agonist, this GLP-1R/GIPR co-agonist has the potential to be an effective weight-loss drug.

The acylated GLP-1R/GIPR co-agonist was also investigated in cynomolgus monkeys. Monkeys were given a single 10 nmol/kg injection of the acylated co-agonist, and 24 h later, a dextrose infusion, during which blood glucose and insulin were measured. The co-agonist lowered blood glucose and increased insulin, both to a greater extent than a matched dose of liraglutide (Finan *et al.* 2013).

The PEGylated co-agonist has even been investigated in humans. In a cohort of healthy, non-diabetic human subjects, a single injection of 4, 8 or 16 mg of the PEGylated co-agonist was followed by a dextrose infusion 72 h later. The co-agonist decreased blood glucose and increased plasma insulin concentration (Finan *et al.* 2013). In more a chronic study, 53 patients with type 2 diabetes were given weekly injections of 4, 12, 20 and 30 mg of the PEGylated co-agonist, for 6 weeks. The co-agonist lowered HbA1c in a dose-dependent manner (Finan *et al.* 2013). The co-agonist was well tolerated, with only mild-to-moderate side effects (Finan *et al.* 2013). A further 13-week Phase II study investigated this compound in patients with type 2 diabetes, with comparisons to placebo and liraglutide treatment. Compared to placebo, treatment with once-daily subcutaneous injections of 1.8 mg of the acylated co-agonist resulted in significant decreases in plasma HbA1c, significant decreases in both fasting and self-reported plasma glucose and a decrease in body weight that was significant at week 8 but not at week 12 (Frias *et al.* 2017). Furthermore, treatment with the acylated co-agonist resulted in a significant reduction in total cholesterol, along with a trend in reduction of LDL, triglycerides, free fatty acids and apolipoprotein B (Frias *et al.* 2017). In the same study, treatment with liraglutide did not result in a change in cholesterol (Frias *et al.* 2017). Decreases in plasma leptin (22% relative to placebo) were also observed (Frias *et al.* 2017), suggesting an increase in leptin sensitivity. In a meal tolerance test, treatment with the compound significantly reduced 2 h post-prandial glucose (Frias *et al.* 2017). In terms of safety, there were no serious adverse effects related to treatment. Reported adverse effects were mostly mild to moderate, and the majority were GI-related events (Frias *et al.* 2017). In addition to these co-agonists, many other GLP-1R/GIPR coagonists are currently in development (Table 1). Whether the promising preclinical results translate into clinical weight-loss benefits remains to be seen.

## GLP-1/GIP/glucagon tri-agonist

**Figure 4:**
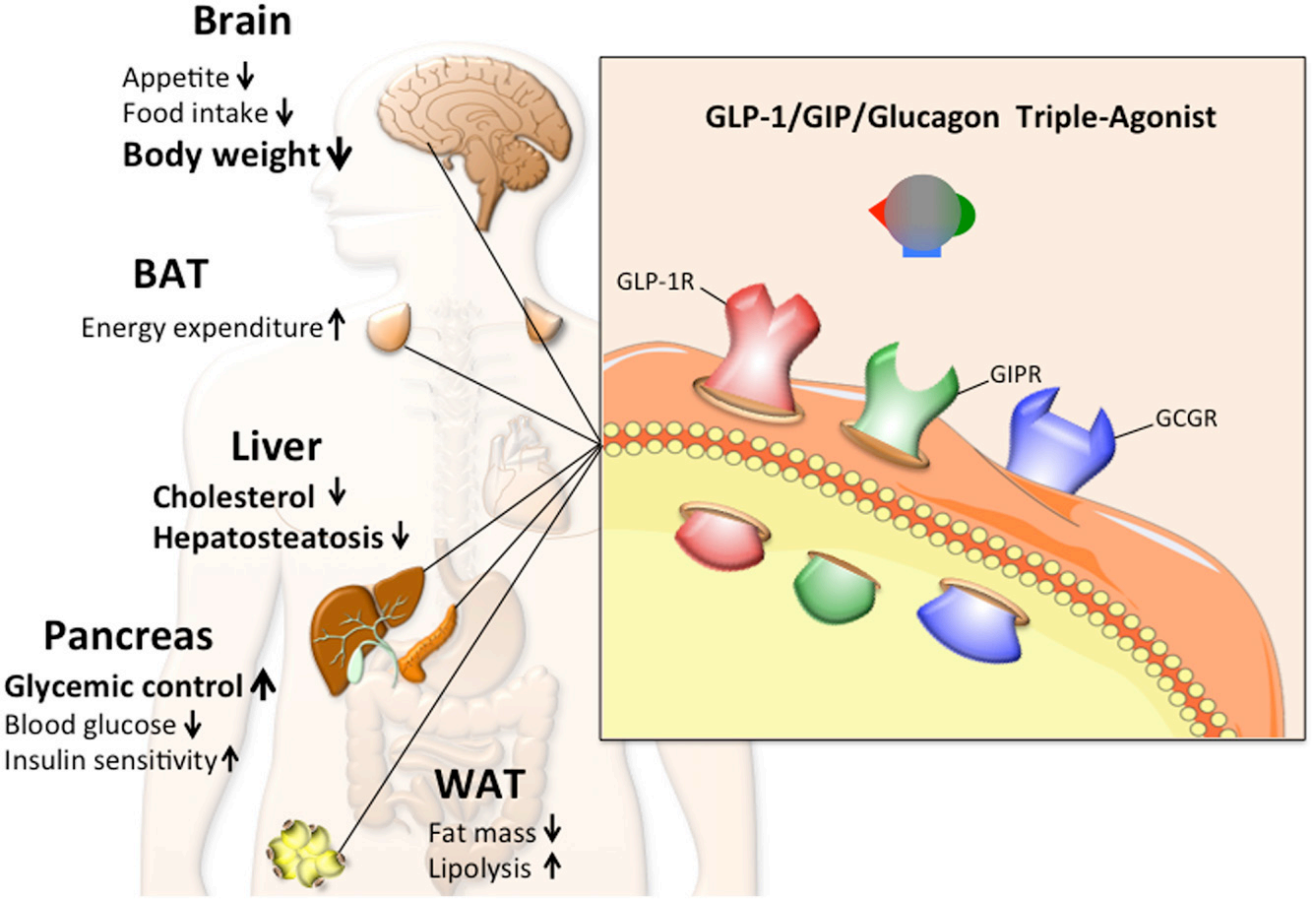
Schematic demonstrating the working principle, metabolic effects and key target tissues of the GLP-1/GIP/glucagon triple agonist, with the size of the text weighted to indicate the magnitude of the observed effect. Arrows pointing upwards indicate an increase or improvement, while arrows pointing downwards indicate a decrease. The triagonist most predominately affects body weight, glycemic control and liver cholesterol and hepatosteatosis.

The preclinical results of the dual GLP-1-based agonists naturally suggest the combination of all three peptides as a potential unimolecular therapy. It was hypothesized that the dual insulinotropic effect of GLP-1 and GIP would optimally buffer against the diabetogenic liability of glucagon while combined agonism at the receptors for GLP-1 and glucagon would restrain any potential obesogenic effect of GIP. The ultimate result of such triple agonism was a profound ability to decrease body weight and to improve glycemic control (Fig. 4).

Beginning with a previously validated GLP-1/glucagon dual agonist sequence, GIP residues were introduced stepwise to create a peptide with equal *in vitro* potency at all three receptors and with superior potency relative to all three native peptides (Finan *et al.* 2015*a*,*b*). This peptide also included an Aib residue at position 2 to protect against DPP-IV cleavage and a C16:0 palmitic acid at the Lys10 position to prolong *in vivo* action (Finan *et al.* 2015*a*,*b*). In DIO mice, a 20-day study of daily subcutaneous injections of as little as 3 nmol/kg of the triple agonist resulted in a 26.6% body weight reduction, which was primarily the result of a loss of fat mass (Finan *et al.* 2015*a*,*b*). In addition, the triple agonist lowered *ad libitum* blood glucose, improved glucose tolerance and lowered circulating insulin levels (Finan *et al.* 2015*a*,*b*), suggesting improved insulin sensitivity. The triple agonist also lowered hepatic lipid content (Finan *et al.* 2015*a*,*b*), which would be beneficial in a translational setting for patients with fatty liver disease and non-alcoholic steatohepatitis (NASH). Importantly, the metabolic benefits of the triple agonist are dependent on signaling at all three target receptors (Finan *et al.* 2015*a*,*b*), demonstrating that it is truly the *triple* agonism responsible for the observed benefits. The efficacy of the triple agonist has also been investigated in female mice. The triagonist was equally efficacious in lowering body weight in DIO female mice compared to fat mass matched male mice (Jall *et al.* 2017). In addition, with a daily dose of 10 nmol/kg for 27 days, the triagonist largely resolved the hepatosteatosis observed in the female mice (Jall *et al.* 2017). Unsurprisingly, the triagonist had only mild effects on glucose tolerance in female mice, since female mice are inherently protected against the development of hyperglycemia and hyperinsulinemia. However, the triagonist did resolve the mild hyperinsulinemia observed in the female mice (Jall *et al.* 2017). Taken together, these results suggest that the triagonist has translational potential in both sexes.

Other triple GLP-1R/GCGR/GIPR agonists are in development (Table 1). Hamni Pharmaceuticals has developed a glucagon-based triple agonist, HM15211, with equal potency at all three receptors *in vitro* (Choi *et al.* 2017, Kim *et al.* 2017). This triple agonist lowers body weight in DIO mice to a greater extent than liraglutide alone and also improves lipid metabolism and hepatic steatosis (Choi *et al.* 2017, Kim *et al.* 2017).

A third example, Syn-GIP-ZP, is a triple agonist created by fusing a GLP-1R/GCGR dual agonist and a GIP analog to the heavy and light chains of Synagis, an antibody with low immunogenicity in humans (Wang *et al.* 2016). This fusion peptide has agonism at all three receptors (Wang *et al.* 2016) and demonstrates that multiagonism is not necessarily limited to structurally related peptides, but can be achieved through fusion to larger biomolecules. Naturally, the advantages of this approach are the increased synthetic flexibility and enhanced pharmacokinetics; however, these molecules must be carefully engineered for stability and carefully designed so that the ratio of agonism between components is metabolically beneficial.

## Are multiagonist peptides the golden therapy for obesity?

Until now, most anti-obesity drugs have been focused either on singular molecular targets or their loose combination in a co-mixture. Unfortunately, none of these strategies has so far led to satisfactory results. While most historic pharmacotherapies are hampered by an unfavorable imbalance between efficacy and safety, this new class of multiagonist drugs has emerged with candidates that may finally close the gap between the efficacy seen with bariatric surgery and pharmacology. Whereas these multiagonist peptides outperform available best in class drugs to treat obesity, only time will tell if they really represent an appreciable step forward. The available preclinical data are encouraging. However, whether the efficacy and tolerability that has been demonstrated in rodents and monkeys also translates to humans remains to be seen. More long-term studies and outpatient trials are required to determine sustainability and safety. While a final judgment requires more long-term clinical studies, we can be carefully optimistic that this new class of specially engineered drugs is lighting the path to a new era in weight-loss pharmacology.

## Declaration of interest

S J B, M K and T D M declare that there is no conflict of interest that could be perceived as prejudicing the impartiality of this review. M H T is a scientific advisor for Novo Nordisk and Erx Biotech.

## Funding

This work was supported in part by funding to M H T from the Alexander von Humboldt Foundation, the Helmholtz Alliance ICEMED & the Helmholtz Initiative on Personalized Medicine iMed by Helmholtz Association and the Helmholtz cross-program topic ‘Metabolic Dysfunction.’ This work was further supported by grants from the German Research Foundation DFG-TS226/1-1, DFG-TS226/3-1, European Research Council ERC AdG HypoFlam no. 695054 and the German Center for Diabetes Research (DZD e.V.). 

## Acknowledgements

The figures were made using material provided by Servier Medical Art (Servier), under the terms of the Creative Commons Attribution 3.0 Unported License.

## Author contribution statement

S J B and T D M conceptualized the project and wrote the manuscript. M K and M H T co-conceptualized the manuscript and edited the manuscript.
